# Complications in the Treatment of Delayed Union and Underlying Chronic Osteomyelitis After Right Crural Fracture Treated With Anterolateral Thigh Flap and Double-Barrelled Vascularized Fibula Graft

**DOI:** 10.7759/cureus.17923

**Published:** 2021-09-13

**Authors:** Sjaak Pouwels, Frank De Jongh, Wouter F Willems, Thuan Nguyen, Steven J Rhemrev

**Affiliations:** 1 Intensive Care Medicine, Elisabeth-Tweesteden Hospital, Tilburg, NLD; 2 Plastic Surgery, Haaglanden Medical Centre, The Hague, NLD; 3 Trauma Surgery, Haaglanden Medical Centre, The Hague, NLD

**Keywords:** trauma surgery, plastic surgery, chronic osteomyelitis, anterolateral thigh flap, masquelet technique

## Abstract

Background: Segmental bone defects pose a major, unsolved clinical challenge and may be the result of high-energy trauma, infection, and tumour resection or revision surgery. Several options exist to reconstruct, including Ilizarov bone transport, Masquelet technique, cylindrical mesh technique, allografts, and vascularized bone autografts. We present a patient with a delayed union of the tibia with concomitant chronic osteomyelitis treated with anterolateral thigh (ALT) flap and double-barrelled vascularized fibula graft.

Case presentation:A 60-year-old male with a chronic pretibial wound with underlying osteomyelitis of the right leg presented himself at the emergency department and was admitted to the surgical ward. He had complex chronic osteomyelitis of a tibial non-union after an earlier right crural fracture (a previous work-related accident). He was treated with an ALT flap and double-barrelled vascularized fibula graft, which was complicated with an additional fracture and breakage of osteosynthesis material.

Conclusion: Segmental bone defects pose a major, unsolved clinical challenge in orthopaedic, trauma-surgical, and plastic surgical practice. Concomitant infections and fractures can be part of the postoperative course. Patients with complex segmental bone defects need to be treated by a multidisciplinary team including at least an (orthopaedic) trauma surgeon, a plastic surgeon, and an infectiologist.

## Introduction

Segmental bone defects pose a major, unsolved clinical challenge in orthopaedic, trauma-surgical, and plastic surgical practice [1-6]. Bone defects may be the result of high-energy trauma, infection, and tumour resection or revision surgery [1,3,4,7]. Several options exist to reconstruct, including Ilizarov bone transport [8-11], Masquelet technique [12], cylindrical mesh technique [13], allografts [14], and vascularized bone autografts [15-17]. Vascularized bone grafting has been recommended for bone defects larger than 6-8 cm [15, 16].

In this case study, we present a patient with a delayed union of the tibia with concomitant chronic osteomyelitis treated with anterolateral thigh (ALT) flap and double-barrelled vascularized fibula graft.

## Case presentation

A 60-year-old male with a chronic pretibial wound with underlying osteomyelitis of the right leg presented himself at the emergency department and was admitted to the surgical ward of the Haaglanden Medical Centre in October 2018. His medical history included the following:

1) In February 2006, the patient's right leg was operatively lengthened using a monotube (due to a length difference between the left and right leg that caused significant complaints while walking). Monotube material was removed in March of the same year and the fracture was fixated using screws. Screws and other osteosynthesis material were removed in 2008 and 2010 after good consolidation of the fracture; 2) In 2012, the patient was treated with antibiotics (flucloxacillin) for a cellulitis infection of the right leg; and 3) He sustained a right crural fracture, which was treated with external fixation and later, after approximately 7 days, an intramedullary nail. This was complicated with osteomyelitis and a break of the intramedullary nail. The osteomyelitis was treated with antibiotics (ceftazidime/clindamycin) and a new intramedullary nail was inserted.

**Figure 1:**
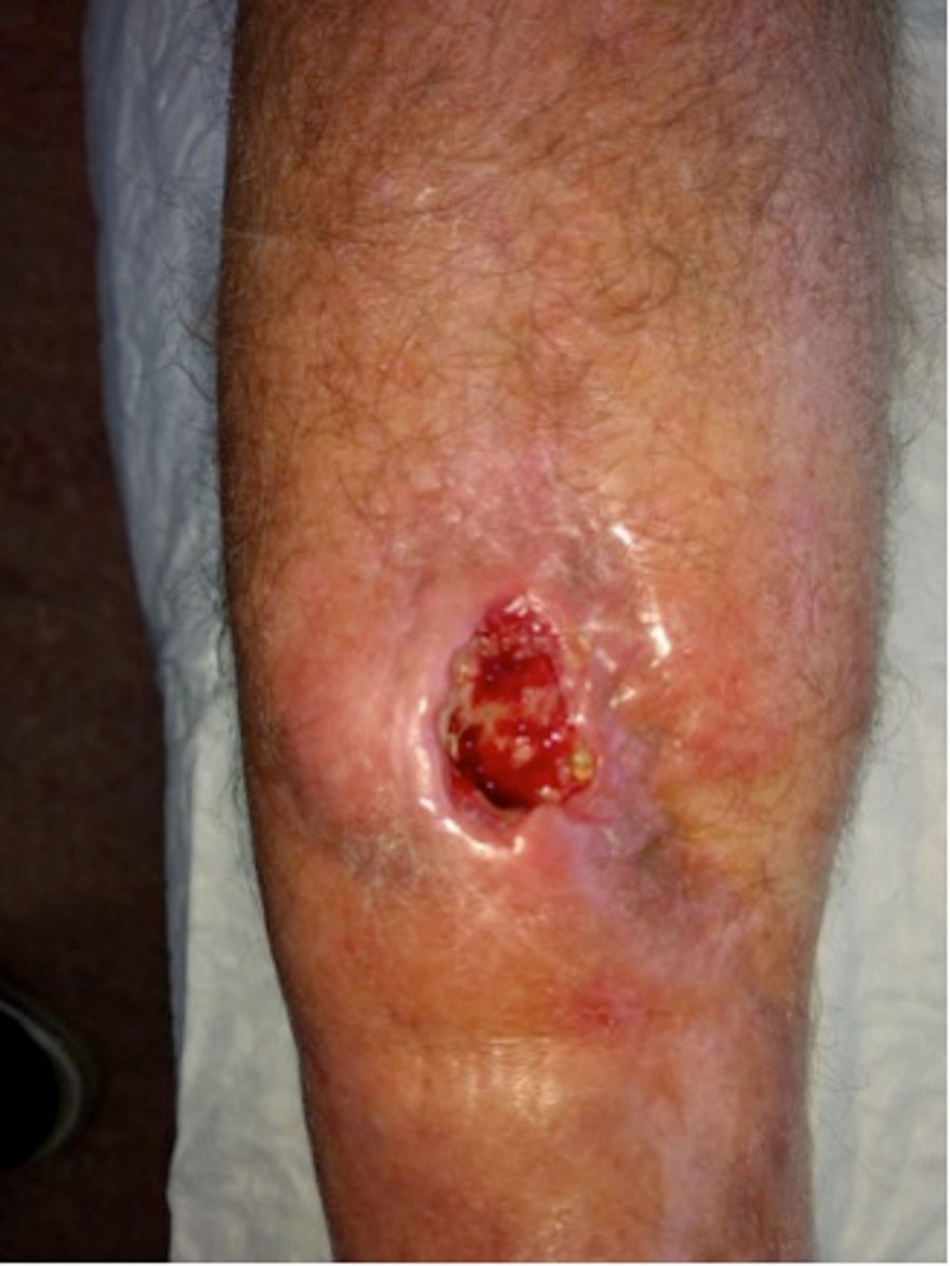
Initial pretibial wound of the right leg (October 2018)

In 2018, the patient presented with a pretibial skin defect of 2x2 cm. Bone is visible caudally in the wound. (Figure 1)

**Figure 2:**
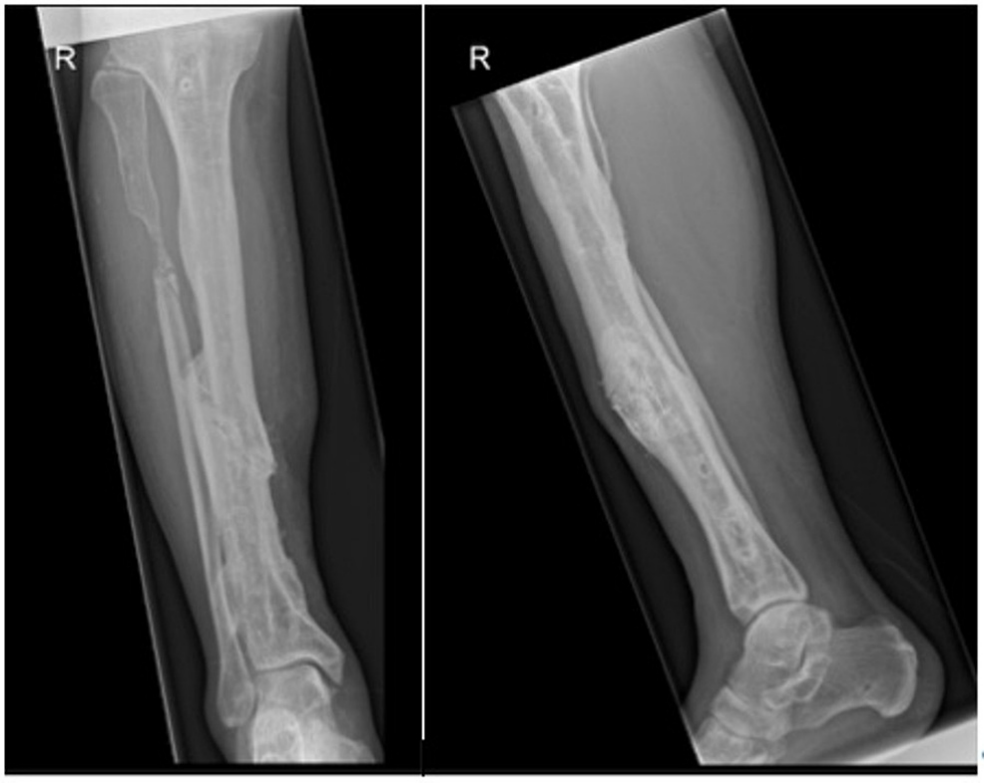
X-ray images showing a non-union of the right tibia

No erythema of the skin or pus was seen. The patient underwent surgery to remove the protruding bone and to clean the wound. Deep cultures of the bone were taken during surgery. The underlying soft tissue was well perfused and postoperatively he was treated with intravenous antibiotics (ceftazidime and clindamycin). In November 2018, the patient returned with pus outflow out of the wound and severe erythema of the wound edges. X-ray images showed and non-union (Figure 2 ). A leucocyte scan (MRI) was performed revealing chronic osteomyelitis around the original fracture with intra-medullary expansion; no pus collections were seen.

**Figure 3:**
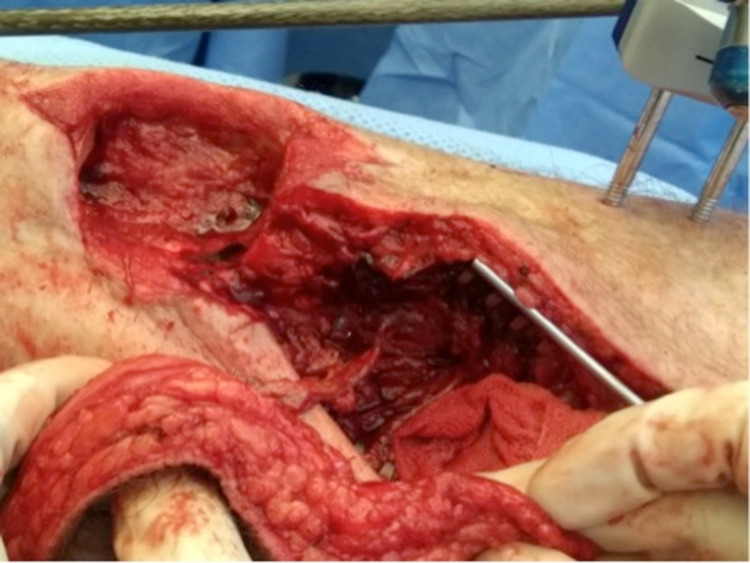
Placement of the anterolateral thigh (ALT) free flap connecting to the dorsal pedal artery (March 2019)

**Figure 4:**
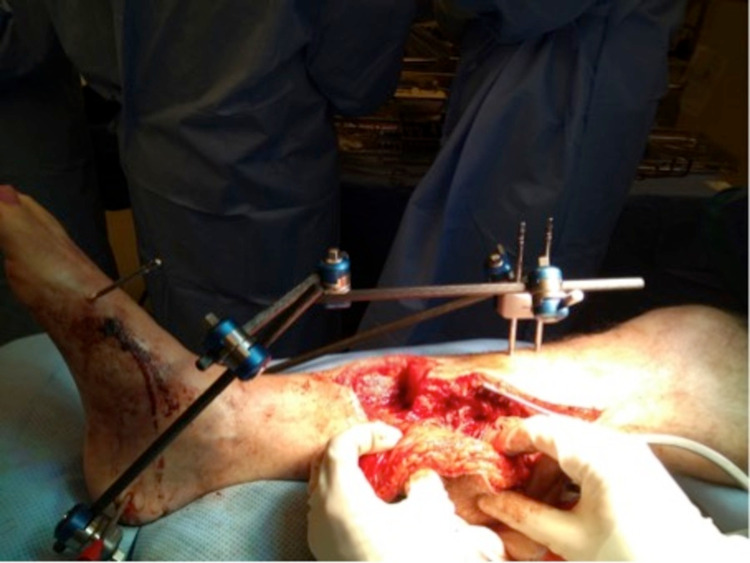
Placement of the anterolateral thigh (ALT) free flap connecting to the dorsal pedal artery (March 2019)

**Figure 5:**
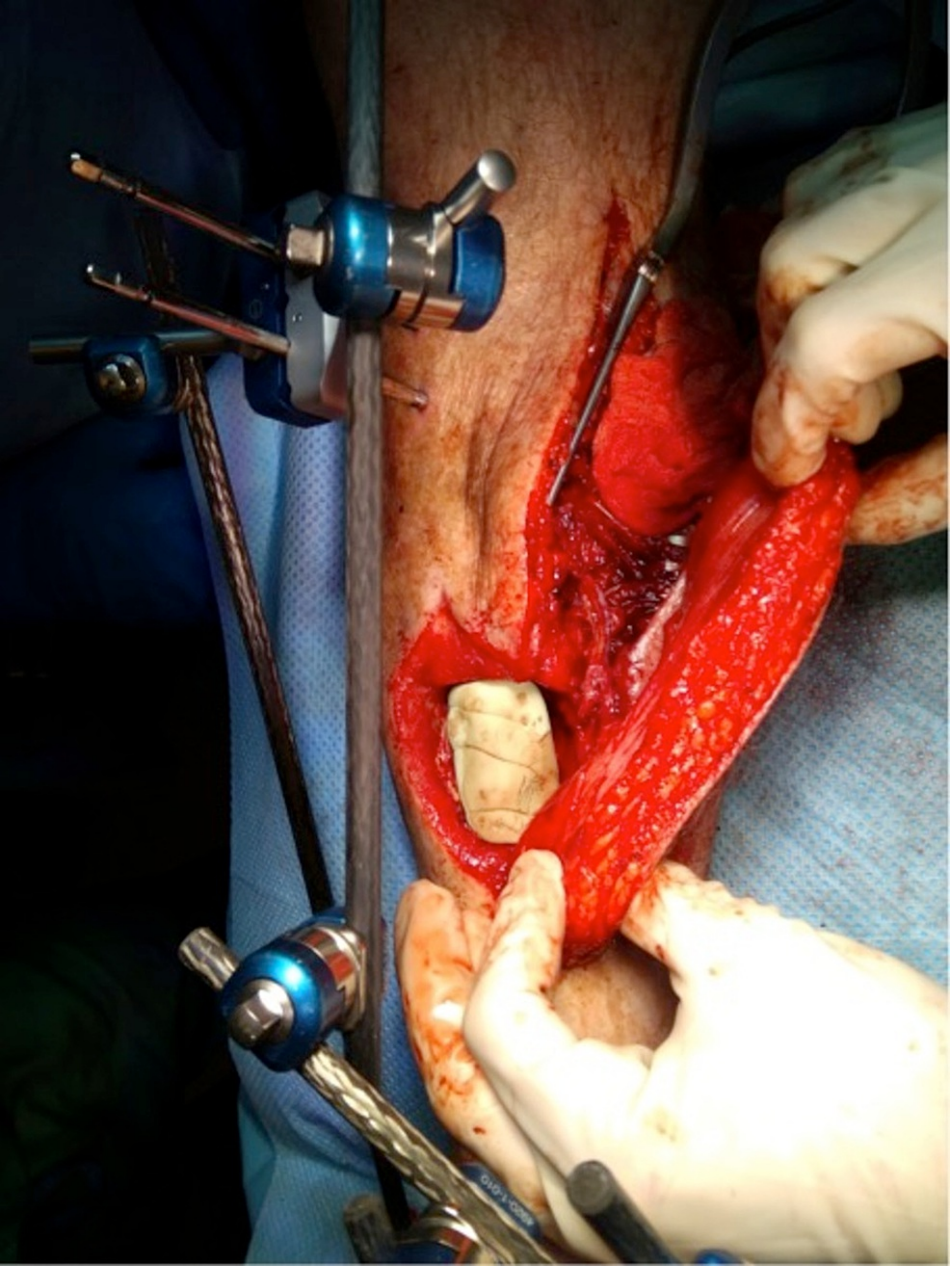
Placement of the anterolateral thigh (ALT) free flap with the cement spacer placed in the tibia defect (March 2019)

**Figure 6:**
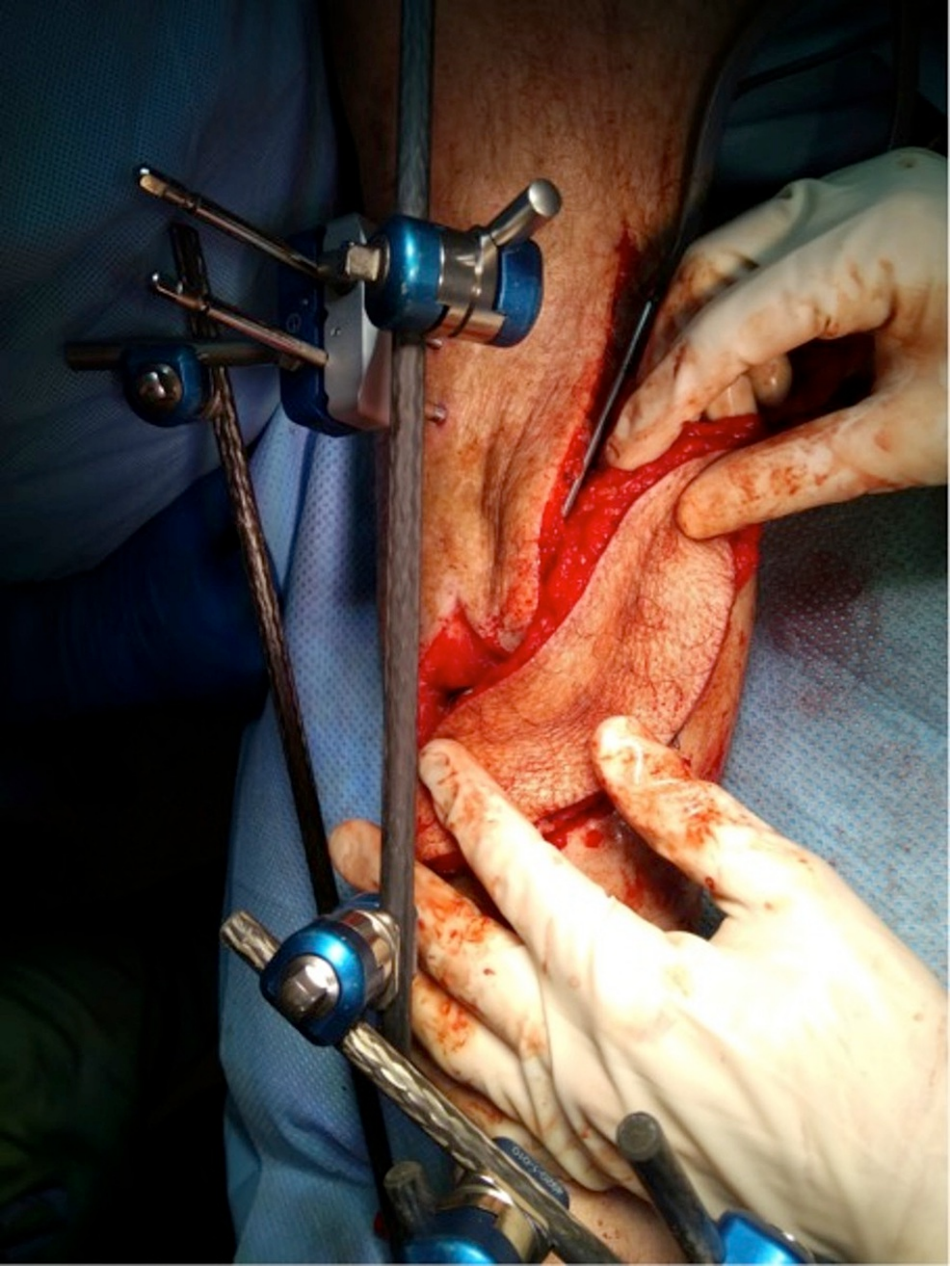
Placement of the anterolateral thigh (ALT) free flap (March 2019)

After several months of antibiotic treatment and vacuum assisted closure (VAC), only a mediocre healing trend was observed. Therefore, in March 2019, the decision was made to remove all of the affected bone, scar tissue, and overlying skin, and to place a gentamycin cement spacer. Pre-operatively the patient stopped smoking and alcohol to minimise the risk of further infection. To cover the fracture and wound, the plastic surgeon was consulted to place an ALT free-flap of the left leg, connected to the dorsal pedal artery. Finally, the surgeons placed an external fixation to stabilize the fracture. The process is seen in Figure 3, Figure 4, Figure 5, and Figure 6.

**Figure 7:**
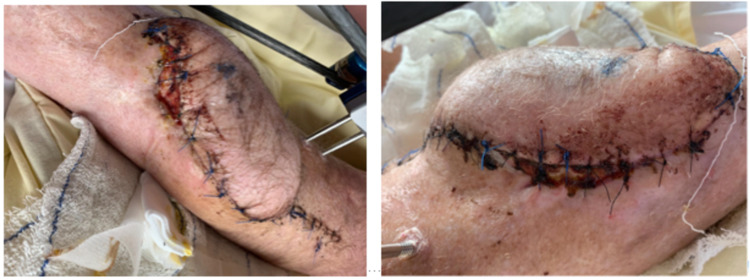
Anterolateral thigh (ALT) free flap two weeks after surgery

Postoperatively, the patient was treated with antibiotics and was kept on bed rest with his leg held at hip height for six days. After this, he started ‘dangling’ with his right leg for 10 minutes twice a day, increasing with 10 minutes every day. Figure 7 shows the postoperative result at two weeks.

**Figure 8:**
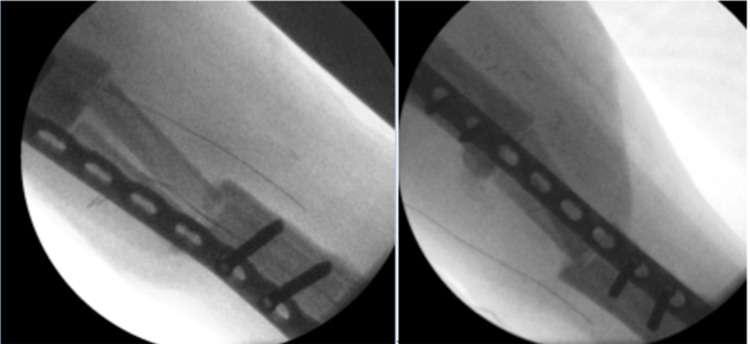
Intraoperative x-ray images of the fibula double barrel placement (April 2019)

In April 2019, the gentamycin cement spacer was removed and the fibula was disconnected proximally at 8cm and distally at 6cm. The distal and proximal ends of the tibia were cleaned and were checked for vascularisation. The fibula was then sawed in half (2x5.5cm) and was placed as a double barrel in the tibia defect. The fibula graft was well vascularized. A locking compression plate (LCP) was placed under x-ray guidance to fixate the tibia. The skin of the lateral lower leg was fixated with a split skin graft (SSG) of the medial upper left leg. This surgery was done by a plastic surgeon together with a trauma surgeon. Intraoperative x-ray images are shown in Figure 8. Cultures of the infected tibia (taken during surgery) showed *Streptococcus constellatus* and bacteroides and the pathology report showed less vital bone with fibrosis and marrow inflammation; no malignancies were found.

Postoperatively, the patient received bed rest for two weeks. An upper leg cast was given for these two weeks, to prevent flexion of the knee and ankle, and thereafter a lower leg cast was used. During this time, the patient received antibiotics (piperacillin/tazobactam, total of six weeks). Three weeks after the operation the patient was discharged and then started ‘dangling’ with the same schedule as before. The patient recovered well and started physiotherapy. In August 2019, the patient was readmitted with a recurrent infection of the right lower leg and spontaneous wounds at the medial and distal site of the previous operations. Antibiotics were again administered. Perioperatively an abscess was incised and drained.

**Figure 9:**
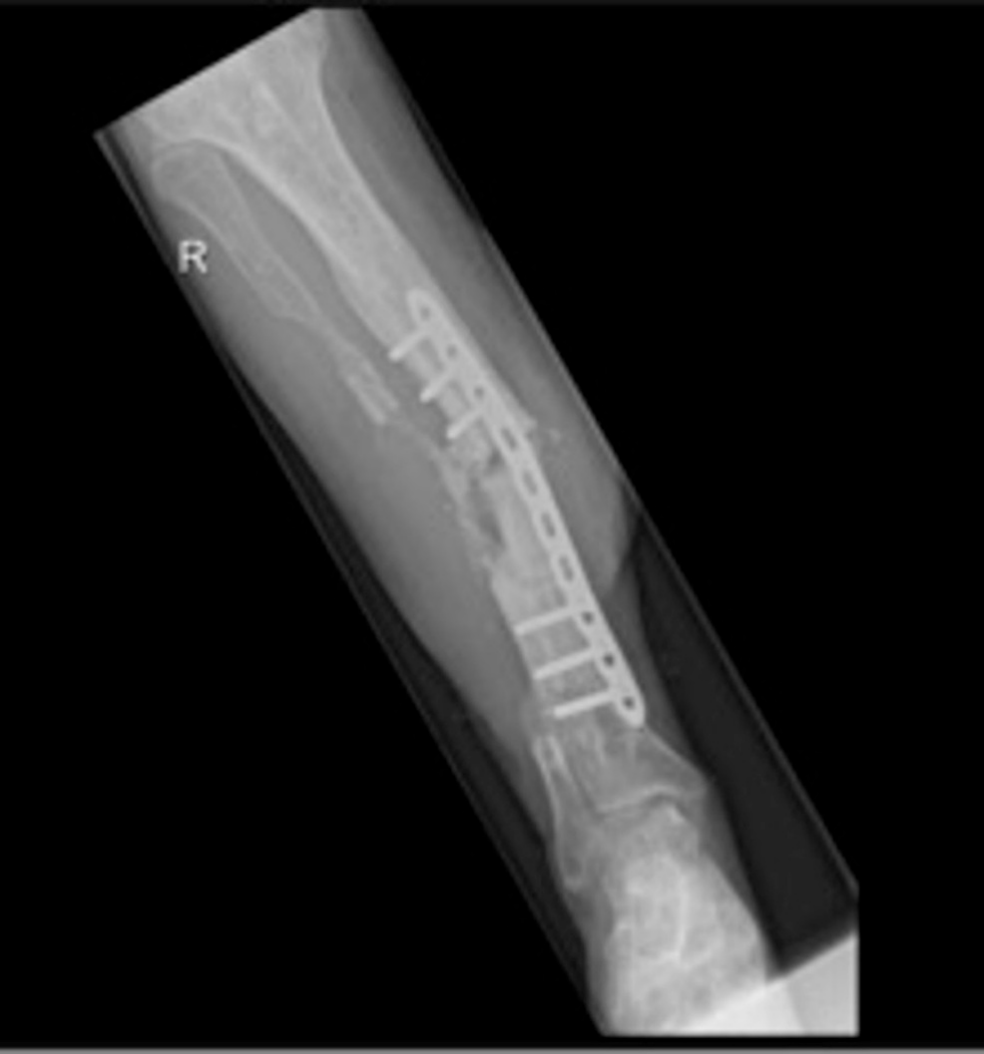
Fracture of the proximal tibia-double barrel fibula transition and break of the locking compression plate (LCP) (November 2019)

In November 2019, an LCP break and fracture of the proximal tibia was found on x-ray after the patient heard a disturbing sound while walking (Figure 9).

**Figure 10:**
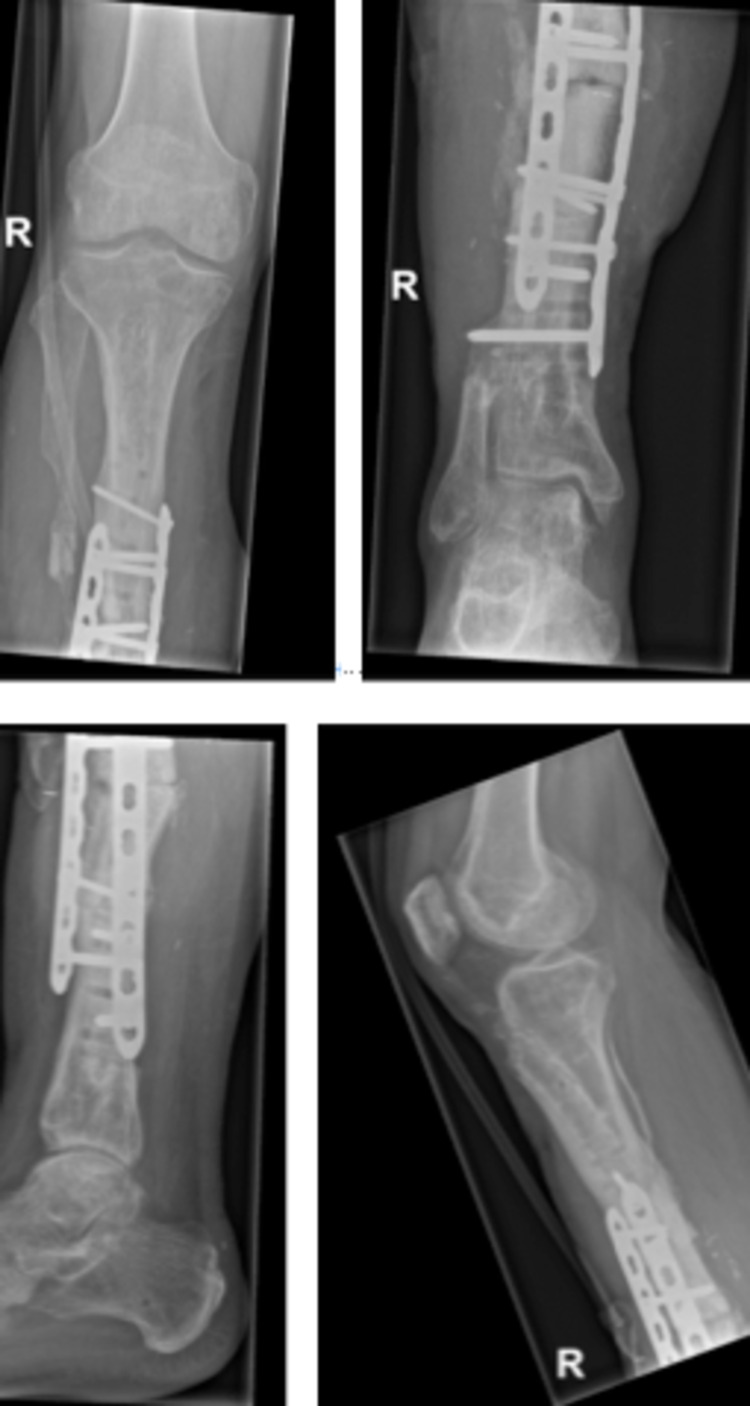
Removal of broken locking compression plate (LCP) and nettoyage of fracture and double-plating using LCP (November 2019)

The wound at the medial side of the ALT flap was circumcised and removed. Incision was made over the old scar at the lateral end (since the ALT vascularisation was at the medial side). Scar tissue, along with the LCP and screws, was removed. At the proximal end of the fibula double barrel, a non-union was seen, distally consolidation was seen. An LCP was placed more medial to secure the tibia and a second LCP was placed for lateral fixation (Figure 10).

**Figure 11:**
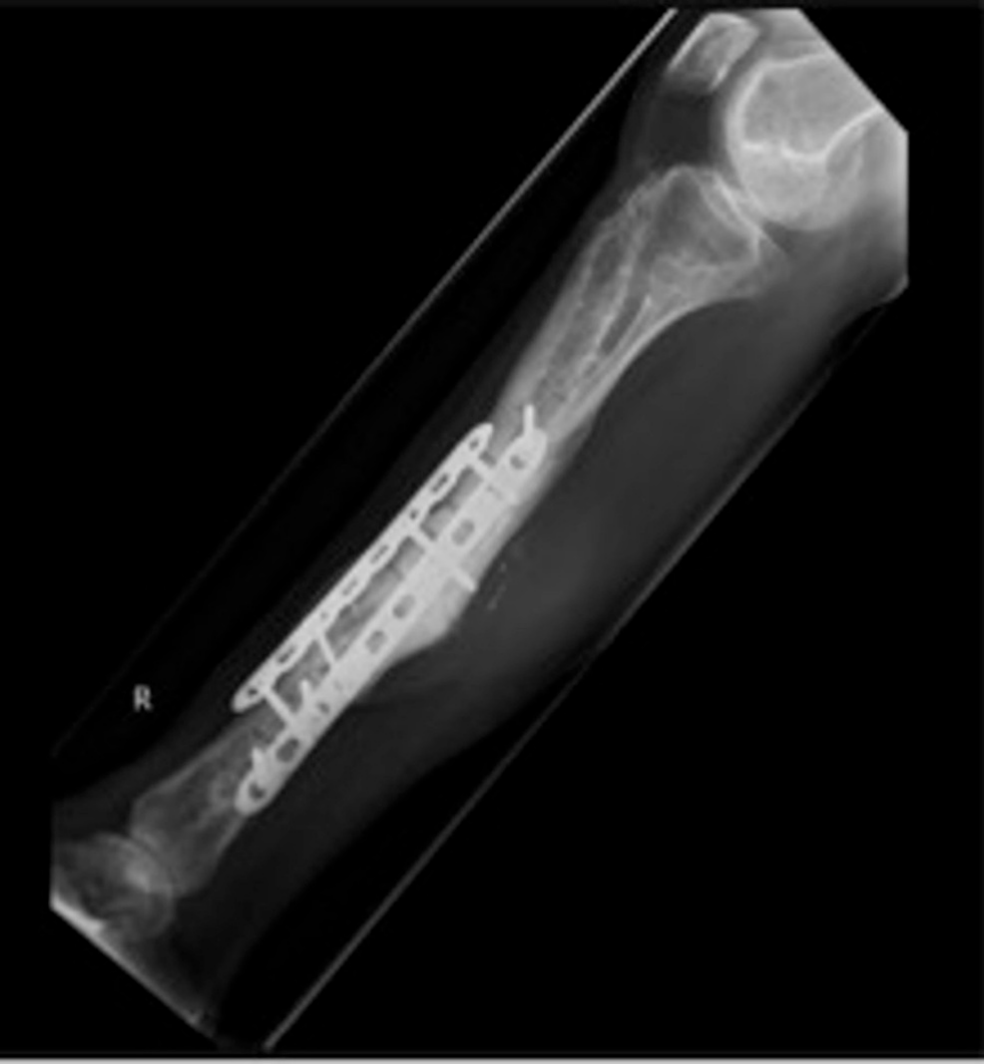
Consolidation of locking compression plate (LCP) (February 2020)

Because of the necessary skin and tissue excision, it was not possible to close the skin primarily and, therefore, a VAC system was again placed. The VAC system was changed in the operation theatre five times to prevent plate infection and since it was too painful in the patient ward.The cultures showed *Staphylococcus haemolyticus* (commensal flora) and the culture of the LCP showed *Enterococcus faecium* and was treated accordingly with levofloxacin and vancomycin for two weeks. Afterwards, the patient received VAC changes at the outpatient clinic two times a week. As of February 2020, the wound is fully recovered with a full consolidation of the LCP (Figure 11). The patient has completely stopped smoking and is back to work again.

## Discussion

The primary bone union rate expected for the bone graft techniques is 91%. In the literature, compression in the non-union site of cancellous graft in non-union areas in the bone is variously reported, also due to additional operations such as the change of a broken implant [18].

In this case, our patient required and still requires, additional operations because of a broken LCP due to recurrent infection, including a formed abscess, after only four months. The events of this case imply that the infection remained after the initial operations, the cause of which we can only speculate. It is possible that, given the long period since the initial fracture, the infection spread throughout the entire marrow and thus was not completely treated by just dissecting the visible infected bone. Also, the choice of initial antibiotics (without culture results) might have influenced the clinical course.

Antonini et al. [19] achieved bone union in 15 out of 18 patients using vascularized fibular grafts, all in traumatic fractures with an average duration from the first injury to surgery of 50 months. According to the results of Azi et al. [18], vascularized graft showed a significant decrease of post-treatment infection, although the definition of infection varied, and different surgical techniques were used [18]. Most studies suggest a two-step reconstruction as a standard approach to managing defects in infected bones. Namely an extensive nettoyage, followed by thorough antibiotic treatment, before performing graft surgery [17,20]. Both steps were performed in our case, which might imply that intrinsic factors might play an important role in restoration. Gao et al. [17] found that the first three years are the most important period to achieve fibular hypertrophy in (free) vascularized fibular grafts, which could effectively prevent stress fractures [17].

## Conclusions

Segmental bone defects pose a major, unsolved clinical challenge in orthopaedic, trauma-surgical, and plastic surgical practice. Concomitant infections and fractures can be part of the postoperative course. Patients with complex segmental bone defects need to be treated by a multidisciplinary team including at least an (orthopaedic) trauma surgeon, a plastic surgeon, and an infectiologist.
